# Exploring Scholarship and the Emergency Medicine Educator: A Workforce Study

**DOI:** 10.5811/westjem.2016.10.32636

**Published:** 2016-12-05

**Authors:** Jaime Jordan, Wendy C. Coates, Samuel Clarke, Daniel P. Runde, Emilie Fowlkes, Jacqueline Kurth, Lalena M. Yarris

**Affiliations:** * Harbor-UCLA Medical Center, Department of Emergency Medicine, Torrance, California; † David Geffen School of Medicine at University of California Los Angeles, Los Angeles, California; ‡ Los Angeles Biomedical Research Institute at Harbor-UCLA, Torrance, California; § University of California Davis Medical Center, Department of Emergency Medicine, Sacramento, California; ¶ University of Iowa Hospitals and Clinics, Department of Emergency Medicine, Iowa City, Iowa; || University of California Los Angeles, Department of Emergency Medicine, Los Angeles, CA; # Oregon Health and Sciences University Medical Center, Department of Emergency Medicine, Portland, Oregon

## Abstract

**Introduction:**

Recent literature calls for initiatives to improve the quality of education studies and support faculty in approaching educational problems in a scholarly manner. Understanding the emergency medicine (EM) educator workforce is a crucial precursor to developing policies to support educators and promote education scholarship in EM. This study aims to illuminate the current workforce model for the academic EM educator.

**Methods:**

Program leadership at EM training programs completed an online survey consisting of multiple choice, completion, and free-response type items. We calculated and reported descriptive statistics.

**Results:**

112 programs participated. Mean number of core faculty/program: 16.02 ± 7.83 [14.53–17.5]. Mean number of faculty full-time equivalents (FTEs)/program dedicated to education is 6.92 ± 4.92 [5.87–7.98], including (mean FTE): Vice chair for education (0.25); director of medical education (0.13); education fellowship director (0.2); residency program director (0.83); associate residency director (0.94); assistant residency director (1.1); medical student clerkship director (0.8); assistant/associate clerkship director (0.28); simulation fellowship director (0.11); simulation director (0.42); director of faculty development (0.13). Mean number of FTEs/program for education administrative support is 2.34 ± 1.1 [2.13–2.61]. Determination of clinical hours varied; 38.75% of programs had personnel with education research expertise.

**Conclusion:**

Education faculty represent about 43% of the core faculty workforce. Many programs do not have the full spectrum of education leadership roles and educational faculty divide their time among multiple important academic roles. Clinical requirements vary. Many departments lack personnel with expertise in education research. This information may inform interventions to promote education scholarship.

## INTRODUCTION

Education research is an important component of the advancement of any medical discipline, and recent publications have outlined a need for initiatives to improve the quality of education studies and support educators who wish to approach educational challenges, questions, and theory in a scholarly manner.^1^–^9^ Medical educators have reported being limited by the following: 1) time to develop and maintain research skills and engage in all phases of the research process; 2) funding to support time and provide research resources; 3) access to expertise for study design and statistical analyses; 4) access to mentors, both within and outside of emergency medicine (EM); and 5) a sense that education research does not result in extrinsic or intrinsic reward in our current educator paradigm.^10^–^12^ However, there is a gap in our knowledge of how EM educators perceive these barriers, and what solutions would be most helpful to them in achieving their education research goals.

Although workforce studies have described the landscape of emergency physicians in general, little is known about what the academic model looks like for EM educators, and how much variability may exist between departments. 13,14 The Council of Emergency Medicine Residency Directors (CORD) Education Scholarship Task Force and CORD Academy for Scholarship in Education in Emergency Medicine recommended that the EM education research community analyze the specific needs of EM educators in a rigorous workforce study and needs assessment. Understanding the job descriptions, available resources and staffing for conducting their educational missions, and the needs of the EM educator workforce is a crucial first step to designing and implementing interventions that will improve the quality of education research and scholarship in EM. The purpose of this study was to illuminate the current workforce model for the academic EM educator.

## METHODS

### Study Setting and Participants

We identified EM residency training programs through the Society of Academic Emergency Medicine (SAEM) residency directory.^15^ One member of the program leadership from each program was invited to participate in the study based on available contact information with preference for program director over assistant/associate program director over program coordinator. We collected data between April 2015 and October 2015.

This study was deemed exempt by the institutional review board of Los Angeles Biomedical Research Institute at Harbor-UCLA Medical Center.

### Study Design

This was a prospective mixed-methods survey. We identified contact information for potential participants through the SAEM residency directory,^15^ programs’ individual websites, and personal knowledge by study team members. Subjects were invited to participate by email and provided with a link to an Internet-based survey. Two follow-up email invitations were sent at weekly intervals to non-responders. Informed consent was implied by those subjects who chose to click on the survey link.

### Instrument

To optimize content validity, the instrument was developed by our study group of EM education researchers with recommendations from members of the CORD Education Scholarship Taskforce according to established guidelines for survey research.^16^ The survey consisted of multiple-choice, completion, and free-response type items. All items were read aloud and discussed among members of the study group to ensure response process validity, and were then piloted with a small group of representative subjects. We made revisions for clarity based on feedback from pilot testing. In order to maximize response rates, incorporate all available data, and preclude guessing answers to unfamiliar queries, completion of all questions on the survey was not required. The survey instrument is available in Appendix A.

### Data Analysis

We calculated and reported descriptive statistics for multiple choice items and completion items with numeric values. Two members of the study team, JJ and WC, independently performed qualitative analysis on the one free-response item using a thematic approach. They examined data line by line to identify recurring concepts and to assign codes, which were then further refined into themes using the constant comparative method.^17^ Discrepancies were resolved by discussion and negotiated consensus to establish a final coding scheme, which was applied to all data. A third analyst, LY, using the agreed-upon coding scheme, independently coded the data. The third analyst had an inter-rater agreement of 86.8% with the first two analysts, and disputes were resolved by in-depth discussion.

## RESULTS

A total of 112/158 (71%) allopathic programs completed the survey and their responses were analyzed. Because of a low response rate from osteopathic programs 9/25 (36%), we excluded their data from analysis. Characteristics of the programs included in the analysis can be found in Table 1.

**Figure 1:**
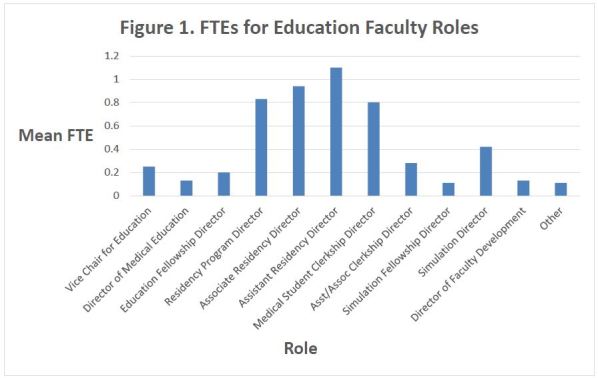
ETEs for educational faculty roles.

**Figure 2:**
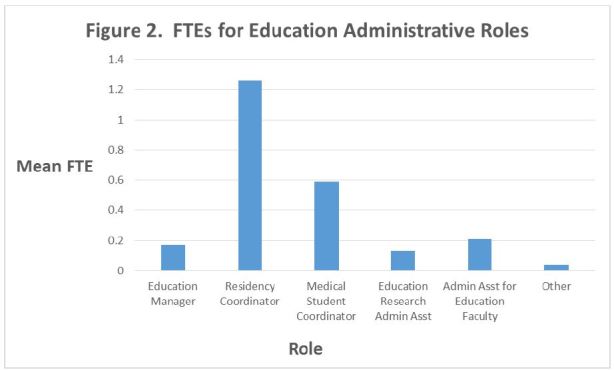
FTEs for education administrative roles.

The mean number of faculty full time equivalents (FTEs) whose primary role is devoted to the educational mission of the program is 6.92 ± 4.92 [5.87–7.98]. These FTEs are distributed among various roles (Table 2 and Figure 1). These faculty have a mean number of 2.34 ± 1.1 [2.13–2.61] administrative FTEs dedicated to education to support them (Table 3 and Figure 2). Few participants took the provided opportunity to write in additional faculty and administrative roles under the “Other” category. For faculty, responses included ultrasound director, other fellowship directors, resident research director, chair, medical school course director, and remediation director. For administration, responses included assistant residency coordinator, assistant fellowship coordinator, and administrative assistant. Because of the limited number of responses, these data were not formally analyzed.

A total of 81/112 (72.3%) programs responded to the question about clinical hours; 67.9% (55/81) reported having an established standard for the number of hours that core faculty, as defined by the Residency Review Committee, work without grant funding or “buy down” from other internal or external sources. This includes faculty from all academic sections. For those who did have an established standard of clinical hours, the mean number of hours/week for core faculty from all academic sections was 26.34 ± 4.64 [25.11–27.58]. When analyzing how base clinical hours for education faculty were determined, major themes that emerged (in descending order of frequency) were determination by academic/administrative position, uniform departmental base standard, individual negotiation, and adherence to Accreditation Council for Graduate Medical Education (ACGME) guidelines. See Table 4 for results of qualitative analysis.

A total of 31/80 (38.8%) programs reported having a designated person in their department with expertise specific to education research study design and statistical analysis. Of these, 35.5% (11/31) have an education research director.

## DISCUSSION

According to our results, a significant portion (approximately 43%) of core faculty are identified by their departments as “education faculty,” or faculty whose primary academic role is devoted to the educational mission of their departments. This likely represents a combination of both perceived importance and practical need. The ACGME requires that EM residency programs maintain a ratio of 1:3 between core faculty and residents.^18^ A critical mass of dedicated faculty is required to run a training program efficiently to develop and implement curricula, assess learners, provide mentoring and advising for trainees, participate in residency selection and clinical competency committees, provide scheduling oversight, and ensure continual quality improvement and program evaluation processes are in place.

The majority of education roles described in our study had mean FTEs of less than one, indicating that departments do not have the full spectrum of leadership positions and/or faculty are serving more than one role. This may create added strain on faculty members who strive to complete the duties of multiple key roles or fill in service gaps potentially without additional protected time or added financial benefit. With this information it is easy to see why time has been identified as a limiting factor to performing education research.^10^ In addition, burnout and attrition are prevalent in academic medicine.^19^ Administrative workload may contribute to burnout, but understanding how educators’ perceptions of the intrinsic reward garnered from their roles is crucial to guide efforts to promote wellness and career satisfaction in academic educators.

Our data also suggest a similar pattern for administrative support staff dedicated to the educational mission of departments. In parallel to the many hats that some education faculty must wear, administrative support staff also appear to perform multiple jobs. It is not clear whether these tasks fall entirely within the realm of education for the administrative staff, or if the departments perceive staff members as purely administrative support rather than an extension of the educational arm of their departments. According to our survey data, the ratio of education faculty to education administrative staff is approximately 3:1. This may represent an additional barrier that educators face in performing scholarship. Without appropriate administrative support, time that faculty could spend on education research and other scholarly endeavors may be diverted to clerical tasks that they must perform themselves. One could argue that this is not the most efficient use of resources.

We found that not all programs have an established standard for clinical hours. Additionally, clinical requirements and how these requirements are determined vary among programs. This may notably contribute to variability in the time and effort that faculty can put towards research and scholarship. It would be interesting to know the degree of impact that clinical load has on scholarly productivity. The mean number of clinical hours/week in this study is approximately 26. If faculty at programs with fewer clinical and administrative hours have a higher degree of scholarly productivity, then this would serve as evidence for allocating more protected time to those who perform education research and scholarship.

We also found that many programs lack personnel with specific methodological expertise in education research, which differs from more traditional research methodology. We postulate that minimal requirements for expertise should include familiarity with qualitative study design as well as familiarity with standard educational formats of hypothesis testing, such as experimental, quasi-experimental, and survey design. This is consistent with current literature citing lack of mentorship and access to expertise in research study design and statistical analysis as barriers.^10^ It is conceivable that existing statistical support faculty could expand their toolkit of knowledge to include these methodologies, or additional faculty who are specifically trained in this area could bridge this gap, especially as education scholarship fellowship graduates become increasingly prevalent.

## LIMITATIONS

Because this was a survey study the results are subject to the limitations inherent to this type of data collection. Additionally, because of the exclusion of osteopathic programs due to poor response rate, the results are limited to allopathic programs. Since we were not able to obtain data from all programs it is possible that additional opinions were not represented. We desired to keep the survey brief to maximize response and in doing so we may have missed important information. We caution readers to consult multiple sources prior to assigning a specific number of hours for each position in their departments. Many questions are still left unanswered. How are departments funding their education mission? What proportion of EM educators conduct education research? Do educators have enough time to fulfill their academic and scholarly responsibilities and does administrative and clinical workload impact scholarly productivity? What rewards do educators receive for their work and how does this affect their wellness and career satisfaction? It will be important to follow up this study with a comprehensive needs assessment of all relevant stakeholder groups.

## CONCLUSION

This study describes the current workforce of EM academic educators and provides further data to support previously identified barriers that educators face in performing scholarship. The results of this study may inform policies and interventions to promote education scholarship and support educators in their academic careers.

## Supplementary Information



## Figures and Tables

**Table 1:** Program characteristics in a study of emergency medicine educator workforce.

	n (Total n=112)
Location	
West	19
Southwest	10
Midwest	29
Southeast	21
Northeast	33
Duration of training	
3 years	79
4 years	33
Number of residents	Mean= 38.95 ± 13.96 [36.36–41.53]
Number of core faculty	Mean= 16.02 ± 7.83 [14.53–17.5]

**Table 2:** Mean full time equivalent (FTE) for faculty education roles.*

Role	Mean FTE ± SD [95% CI]
Vice chair for education	0.25 ± 0.38 [0.17–0.33]
Director of medical education	0.13 ± 0.31 [0.06–0.19]
Education fellowship director	0.20 ± 0.44 [0.11–0.3]
Residency program director	0.83 ± 0.28 [0.77–0.89]
Associate residency director	0.94 ± 0.77 [0.77–1.1]
Assistant residency director	1.1 ± 1.05 [0.87–1.32]
Medical student clerkship director	0.8 ± 0.46 [0.7–0.89]
Assistant/associate clerkship director	0.28 ± 0.44 [0.18–0.37]
Simulation fellowship director	0.11 ± 0.3 [0.05–0.18]
Simulation director	0.42 ± 0.42 [0.33–0.51]
Director of faculty development	0.13 ± 0.31 [0.06–0.2]
Other	0.11 ± 0.52 [0–0.22]

* Note: One individual fulfills one FTE. If an individual fulfills multiple roles, respondents were asked to estimate the portion of FTE that is dedicated to each role.

*FTE*, full time equivalent.

**Table 3:** Mean full time equivalent (FTE) for education administrative roles.*

Role	Mean FTE ± SD [95% CI]
Education manager	0.17 ± 0.36 [0.9–0.25]
Residency coordinator	1.26 ± 0.53 [1.15–1.38]
Medical student coordinator	0.59 ± 0.44 [0.49–0.68]
Education research administrative assistant	0.13 ± 0.36 [0.05–0.21]
Direct administrative assistant for education faculty	0.21 ± 0.47 [0.1–0.31]
Other	0.04 ± 0.17 [0–0.08]

* Note: One individual fulfills one FTE. If an individual fulfills multiple roles, respondents were asked to estimate the portion of FTE that is dedicated to each role.

**Table 4:** Results of qualitative analysis regarding how base clinical hours for education faculty are determined.

Question: Please describe how base clinical hours for education faculty are determined in your department.		

Theme	Number of comments	Example
		
Determination by academic/administrative position	20	“based on teaching responsibilities, hours decreased depending on educational roles”
Uniform departmental base standard	17	“All full time faculty work 10–11 shifts per month, including education faculty. Vice Chairs work 6–7 shifts per month and the chair works 5–6 shifts per month.”
Individual negotiation	15	“The chair sets each faculty base hours on an individual basis. There is no set standard, and no transparency about how the systems works. It can change year to year.”
Accreditation council for graduate medical education (ACGME) guidelines	14	“ACGME maximum”
