# Supplementary material for: Exploring Scholarship and the Emergency Medicine Educator: A Workforce Study
Source: West J Emerg Med. 2016 Dec 5;18(1):163–8. doi: 10.5811/westjem.2016.10.32636 (PMC5226754; doi:10.5811/westjem.2016.10.32636)
Supplement: Supplementary file 1 [file wjem-18-163-s001.docx]

Appendix A. Data Collection Instrument

1. Please select which region your program is located in:

_____West (AK, CA, CO, HI, ID, MT, NV, OR, UT, WA, WY)

_____Southwest (AZ, NM, OK, TX)

_____Midwest (IA, IL, IN, KS, MI, MN, MO, NE. ND, OH, SD, WI)

_____Southeast (AL, AR, DC, DE, FL, GA, KY, LA, MD, MS, NC, SC, TN, VA, WV)

_____ Northeast (CT, ME, MA, NH, NJ, NY, PA, RI, VT)

1. What is the duration of your EM residency program?

_____3 years

_____4 years

1. How many total residents are in your program currently?
2. How many **CORE** full time academic faculty members *(as defined by the Residency Review Committee)* are in your department currently?
3. We are trying to estimate the number of faculty whose primary academic role is devoted to the **EDUCATION MISSION** of the department. How many faculty full time equivalents (FTE) are dedicated to the education mission in your academic department, including medical student, residency, and faculty development roles?*(Please EXCLUDE fellowship directors other than Education and Simulation fellowship directors.)*
4. How many FTEs are devoted to the following roles in your department? If an individual has more than one role, please estimate the portion of FTE that is dedicated to that role *(i.e. 0.5). For example, if 2 faculty members’ sole roles are associate residency director and 1 faculty member splits their time between being the clerkship director and the education fellowship director then list: assistant residency director 2; clerkship director 0.5; education fellowship director 0.5.*

_____ Vice Chair for Education

_____ Director of Medical Education

_____ Education Fellowship Director

_____Residency Program Director

_____Assistant Residency Director

_____Associate Residency Director

_____Clerkship Director

_____Assistant/Associate Clerkship Director

­­­­_____Simulation Fellowship Director

_____Simulation Director

_____Director of Faculty Development

_____Other: ________________________

1. How many full time equivalents (FTE) are dedicated to administrative support for the **EDUCATION MISSION** of the department *(includes education managers, residency coordinators, education fellowship coordinators, education research administrative assistants, etc)*? _______
2. Please indicate how many FTEs are devoted to each role. If an individual has more than one role, estimate the portion of FTE that is dedicated to education *(i.e 0.5)*.

_____ Education Manager

_____ Residency Coordinator

_____ Medical Student Coordinator

_____ Education Research Administrative Assistant

_____ Direct Administrative Assistant for education faculty

_____ Other

1. Is there an established standard for how many clinical hours **CORE** faculty in your department *(in all academic sections)* work, without grant funding or “buy down” from any internal or external source?

_____Yes

_____No

_____Unsure

1. If yes, how many clinical hours/week do CORE faculty in your department (in all academic sections) work, without grant funding or “buy down” from any internal or external source?
2. Please describe how base clinical hours for **EDUCATION FACULTY** are determined in your department.

[Text box]

1. Is there a designated person in your department with expertise specific to **EDUCATION** **RESEARCH** *(research related to student learning, teaching methods, classroom dynamics, etc)* study design and statistical analysis?

_____Yes

_____No

_____Unsure

1. If yes, Do you have an education research director? *(An individual whose academic role is specifically to oversee education research activities)*

_____Yes

_____No

_____Unsure
